# P-881. A Retrospective Review of *Enterobacter* c*loacae* complex Bacteremia: 2019-2023

**DOI:** 10.1093/ofid/ofae631.1072

**Published:** 2025-01-29

**Authors:** Samuel Harder, Maison Z Leitch, Bonita Lee, Stephanie W Smith, William Stokes

**Affiliations:** University of Alberta, Edmonton, Alberta, Canada; University of Alberta, Edmonton, Alberta, Canada; University of Alberta, Edmonton, Alberta, Canada; University of Alberta, Edmonton, Alberta, Canada; University of Alberta, Edmonton, Alberta, Canada

## Abstract

**Background:**

Enterobacter cloacae complex is one of the most common Enterobacterales considered at moderate to high risk for clinically significant AmpC production. AMP-C betalactamases confer resistance to broad spectrum cephalosporins and common beta-lactam betalactamases including Amoxicillin-Clavulanate and Piperacillin-Tazobactam. Carbapenems are often used empirically when this organism is isolated but there is a current gap in the literature regarding in which circumstances non-carbapenem antibiotics can be used safely for patients with enterobacter cloacae complex infections. The aim of this study was to examine independent predictors of mortality for patients with enterobacter cloacae complex bacteremia and identify patients which can appropriately receive non-carbapenem based therapy safely and effectively without negative impacts on mortality.

**Methods:**

We conducted a retrospective analysis of 285 episodes of enterobacter cloacae complex bacteremia. The main outcome measure was mortality at 30 days.The χ2 or Fisher's exact test were used to compare categorical variables. To identify the independent risk factors of infection and mortality, a binary logistic regression model was used to control for the effects of confounding variables.

**Results:**

Of 285 patients 17.5% (50) were deceased at 30 days, 110 patients received a beta-lactam betalactamase inhibitor combination initially and 80 patients received an initial broad spectrum cephalosporin. Of 209 patients for whom antibiotic data was available 73 were changed to a carbapenem within 24 hours of the initial blood culture being drawn. In univariate analysis only source was identified as a statistically significant risk factor for mortality at 30 days. In univariate and multivariate binary logistic regression analysis change to carbapenem within 24 hours of blood culture was not an independent risk factor of death at 30 days.

**Conclusion:**

Change to carbapenem within 24 hours of cultures being taken was not an independent risk factor of 30 day mortality. Further studies are needed to determine in what group broad spectrum cephalosporins or beta-lactam betlactamase inhibitors are appropriate therapy for enterobacter cloacae.

**Disclosures:**

**All Authors**: No reported disclosures

